# Adaptation of forest trees to climate change

**DOI:** 10.1186/1753-6561-5-S7-I13

**Published:** 2011-09-13

**Authors:** Christophe Plomion, Ivan Scotti, Sylvain Delzon, Jean-Marc Gion

**Affiliations:** 1 INRA, UMR BIOGECO, France; 2 INRA, UMR ECOFOG, France; 3 CIRAD, UMR BIOGECO, France

## 

There is an urgent need to determine the adaptive potential of forest trees given their importance in ecosystem functioning and the associated ecological and economic services they provide. Indeed, underestimating rapid adaptation could lead to unnecessary recommendations such as the use of non-native (and perhaps non-adapted) genetic material for reforestation. Overestimating adaptive potential could have detrimental consequences if population decline massively and prove enable to regenerate.

A critical step to study individual’s and specie’s responses to variation in climate is to examine the basis of adaptation (at the phenotypic and molecular levels) under natural conditions and across multiple years. In this context, these sessile and long lived species have emerged as a model system for studying adaptation into the wild. Whether forest trees will be able to adapt in situ fast enough to outstrip the rate of such modifications relative to their longevity is still largely debated and remains an open question.

The objectives of this presentation are three fold:

i/ provide an overview of the concept of ADAPTATION: a process whereby a genotype or a population becomes better suited to its habitat. Adaptation to climate change can occur through phenotypic plasticity (a mechanism by which individuals can withstand large environmental fluctuation without genetic change) and evolution (i.e. response to natural selection pressure when the level of standing genetic diversity or diversity introduced by gene flow permits it).

ii/ provide some clues (from historical and contemporary data) on how closely can forest tree adaptation be expected to track climate change as rapid as envisioned for the future. They have high phenotypic plasticity which allows them to tolerate wide environmental variations during their lifetime. Besides, there are some evidence against the idea that rapid environmental changes overwhelms evolutionary processes preventing adaptation to local environments.

iii/ review how genes that matter for adaptation can be detected using 'omics technology in combination with population and quantitative genetics approaches.

